# Cholesterol Side-Chain Cleavage Gene Expression in Theca Cells: Augmented Transcriptional Regulation and mRNA Stability in Polycystic Ovary Syndrome

**DOI:** 10.1371/journal.pone.0048963

**Published:** 2012-11-14

**Authors:** Jessica K. Wickenheisser, Jessica M. Biegler, Velen L. Nelson-DeGrave, Richard S. Legro, Jerome F. Strauss, Jan M. McAllister

**Affiliations:** 1 Department of Pathology, Pennsylvania State University College of Medicine, Hershey, Pennsylvania, United States of America; 2 Department of Obstetrics and Gynecology, Pennsylvania State University College of Medicine, Hershey, Pennsylvania, United States of America; 3 Department of Obstetrics and Gynecology, Virginia Commonwealth University, Richmond, Virginia, United States of America; Imperial College London, United Kingdom

## Abstract

Hyperandrogenism is characteristic of women with polycystic ovary syndrome (PCOS). Ovarian theca cells isolated from PCOS follicles and maintained in long-term culture produce elevated levels of progestins and androgens compared to normal theca cells. Augmented steroid production in PCOS theca cells is associated with changes in the expression of genes for several steroidogenic enzymes, including *CYP11A1*, which encodes cytochrome P450 cholesterol side-chain cleavage. Here, we further examined *CYP11A1* gene expression, at both the transcriptional and post-transcriptional level in normal and PCOS theca cells propagated in long-term culture utilizing quantitative RT-PCR, functional promoter analyses, and mRNA degradation studies. The minimal element(s) that conferred increased basal and cAMP-dependent *CYP11A1* promoter function were determined. CYP11A1 mRNA half-life in normal and PCOS theca cells was compared. Results of these cumulative studies showed that basal and forskolin stimulated steady state *CYP11A1* mRNA abundance and *CYP11A1* promoter activity were increased in PCOS theca cells. Deletion analysis of the *CYP11A1* promoter demonstrated that augmented promoter function in PCOS theca cells results from increased basal regulation conferred by a minimal sequence between −160 and −90 bp of the transcriptional start site. The transcription factor, nuclear factor 1C2, was observed to regulate basal activity of this minimal *CYP11A1* element. Examination of mRNA stability in normal and PCOS theca cells demonstrated that CYP11A1 mRNA half-life increased >2-fold, from approximately 9.22+/−1.62 h in normal cells, to 22.38+/−0.92 h in PCOS cells. Forskolin treatment did not prolong CYP11A1 mRNA stability in either normal or PCOS theca cells. The 5′-UTR of CYP11A1 mRNA confers increased basal mRNA stability in PCOS cells. In conclusion, these studies show that elevated steady state *CYP11A1* mRNA abundance in PCOS cells results from increased transactivation of the *CYP11A1* promoter and increased CYP11A1 mRNA stability.

## Introduction

PCOS is the most common cause of infertility in women [1] and affects approximately 7% of women of reproductive age. PCOS ovaries are characterized by the accumulation of small follicles 4–7 mm in diameter, with hypertrophied theca interna layers. Reproductive endocrine abnormalities in PCOS include amenorrhea or oligomenorrhea, infertility, hirsutism and acne resulting from increased ovarian androgen production [2]–[6]. Theca cells are recognized as one of the primary sources of excess androgen biosynthesis in women with PCOS [7]–[10]. In response to luteinizing hormone, theca cells express a variety of genes encoding components of the steroidogenic pathway that are necessary for androgen and progestin biosynthesis [11]–[13]. Steroidogenic acute regulatory protein (StAR) promotes the translocation of cholesterol from the outer to the inner mitochondrial membrane [14], [15], where cytochrome P450 side chain cleavage enzyme, P450scc, converts cholesterol to pregnenolone, the first step in steroid hormone synthesis [16], [17]. The synthesis of androgens is also contingent upon the expression of the cytochrome P450 17α-hydroxylase (*CYP17A1*) gene, which encodes a single cytochrome P450 (P450c17) with both 17α-hydroxylase and C17, 20 lyase activities responsible for the conversion of pregnenolone to 17α-hydroxypregnenolone, and subsequently dehydroepiandrosterone (DHEA) [18].

Our previous studies demonstrated that both progesterone and androgen production are persistently elevated in theca cells isolated from the ovaries of women with PCOS, propagated for successive population doublings *in vitro* [19], [20]. This increase in steroid production in PCOS theca cells is associated with augmented expression of several steroidogenic enzymes, including *CYP11A1*, *CYP17A1*, and 3β-hydroxysteroid dehydrogenase, type 2 (*HSD3B2*) [19], [21]. The underlying mechanism of this coordinated upregulation is unknown. Investigation of *CYP17* gene expression in normal and PCOS theca cells has revealed that increased CYP17 mRNA abundance results from both increased transactivation of the promoter and augmented mRNA stability in PCOS [22], [23]. The transcription factor, NF-1C2, was found to play an important role in increased basal *CYP17A1* gene expression in PCOS theca cells and adrenal H295 cells [24]. In addition, the 5′-untranslated (5′UTR) region of CYP17 mRNA was shown to confer increased mRNA half-life in PCOS theca cells as compared to normal theca cells, thus increasing *CYP17A1* expression in both of the above cases.

We previously reported that augmented *CYP11A1* gene expression also involves increased transactivation of the *CYP11A* gene and promoter in PCOS theca cells [25]. In the present study, we have examined the extent to which changes in transcriptional and post-transcriptional regulation play a role in increased *CYP11A1* gene expression in PCOS theca cells. We have identified the boundaries of the *CYP11A1* promoter that confer increased basal and cAMP-dependent expression in normal and PCOS theca cells utilizing functional promoter analyses. Moreover, we have identified the minimal element that confers increased basal regulation in PCOS theca cells. We investigated the possibility that the transcription factor, nuclear factor 1 (NF-1C2), which we had reported to play a role in basal CYP17 gene expression in PCOS theca cells, coordinately regulates basal *CYP11A1* gene expression. In this report, we also performed CYP11A1 mRNA half-life and mRNA degradation studies, to determine the overall contribution of increased CYP11A1 stability to increased gene expression in PCOS theca cells.

## Materials and Methods

### Ethics Statement

Human theca interna tissue was obtained from follicles of women undergoing hysterectomy for non-related purposes, following informed consent under protocol that has been approved by the Institutional Review Board (IRB) of the Human Subjects Protection Office of the Pennsylvania State University College of Medicine. Signed IRB approved consent was obtained prior to surgery at the patient's pre-operative visit. All surgeries were performed for non-related gynecologic indications, most commonly for dysfunctional uterine bleeding and/or intrauterine abnormalities such as endometrial hyperplasia or endometriosis. Oophorectomy involving one or both ovaries, was discussed individually with these women by their physicians, and was chosen as a course of treatment in many cases by the patient to ameliorate pelvic pain and/or menorrhagia.

### Theca cell isolation and propagation

Fragments of ovaries were obtained from discarded ovarian specimens from pathology gross room. Individual follicles were dissected away from ovarian stroma, and dispersed with 0.05% collagenase I, 0.05% collagenase IA, and 0.01% deoxyribonuclease, in medium containing 10% fetal bovine serum (FBS), as previously described [26]. The isolated follicles were size-selected for diameters ranging from 3–5 mm so that theca cells derived from follicles of similar size from normal and PCOS subjects could be compared. Theca cells were cultured on fibronectin coated dishes utilizing previously described “growth medium” (1∶1 mixture of Dulbecco's Eagles Medium (DME) and Hams F-12 medium, containing 5% FBS, 5% horse serum (HS), 2% UltroSer G, 20 nM insulin, 20 nM selenium, 1 µM vitamin E, and antibiotics) [27]. Sera and growth factors were obtained from the following sources: FBS and DME/F12 (Irvine Scientific, Irvine, CA): horse serum (Life Technologies, Grand Island, NY); UltroSer G (Reactifs IBF, Villeneuve-la-Garenne, France): other compounds were purchased from Sigma (St. Louis, MO). The cells were grown in reduced oxygen tension (5% O_2_, 90% N_2_, and 5% CO_2_) and given supplemental antioxidants (vitamin E and selenium) to prevent oxidative damage [27]. The reduced oxygen tension and concentrations of bovine (5%) and horse serum (5%) and UltroSer (2%) in the “growth medium” described above, were previously reported to be the most effective to grow human theca cells for successive population doublings with maintenance of inducible 17α-hydroxylase activity and steroidogenic function [27], [28].

The theca cell cultures utilized in these studies have been described and functionally characterized previously [19], [22], [29], [30]. Experiments comparing PCOS and normal theca were performed utilizing 4^th^-passage (31–38 population doublings) theca cells isolated from size-matched follicles. The use of 4^th^ passage cells allowed us to perform multiple experiments from the same patient population, and were propagated from frozen stocks of second passage cells in the media described above. For all studies, theca cell cultures obtained from at least 5 independent normal and 5 independent PCOS patients were examined unless otherwise specified. The passage conditions and split ratios for all normal and PCOS cells were identical.

The PCOS and normal ovarian tissue came from age-matched women, 38–40 years old. The diagnosis of PCOS was made according to NIH consensus guidelines [31], which include hyperandrogenemia, oligoovulation, polycystic ovaries, and the exclusion of 21-hydroxylase deficiency (AM 17α-hydroxyprogesterone), Cushings (elevated cortisol, and physical examination), and hyperprolactinemia. All of the PCOS theca cell preparations studied came from ovaries of women with fewer than six menses per year and elevated serum total testosterone or bioavailable testosterone levels, as previously described [19], [32]. Each of the PCOS ovaries contained multiple subcortical follicles of less than 10 mm in diameter. The control (normal) theca cell preparations came from ovaries of fertile women with normal menstrual histories, menstrual cycles of 21–35 days, and no clinical signs of hyperandrogenism. Neither PCOS nor normal subjects were receiving hormonal medications at the time of surgery. Indications for surgery were dysfunctional uterine bleeding, endometrial cancer, and/or pelvic pain.

### CYP11A1 mRNA Quantitation

**Figure 1:**
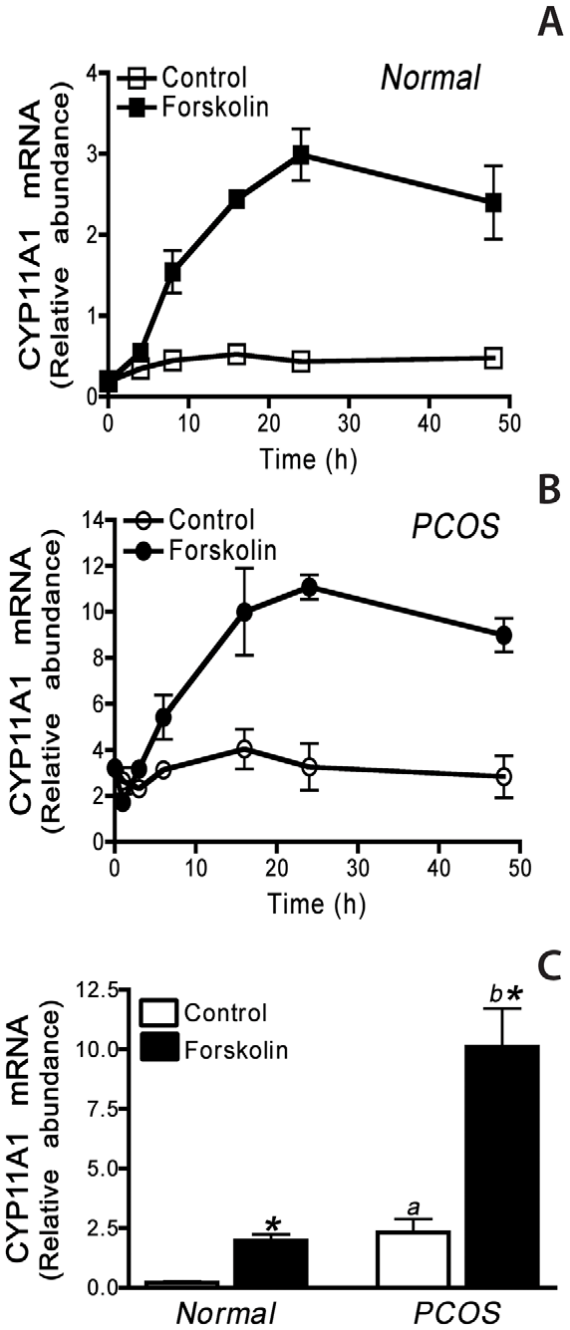
Quantification of *CYP11A1* mRNA abundance in normal and PCOS theca cells. The time course of CYP11A1 mRNA accumulation was examined in **A)** normal or **B)** PCOS theca cells treated in serum free medium for 0, 4, 8, 16, 24, or 48 h in the presence and absence of 20 µM forskolin. **C)** CYP11A1 mRNA accumulation in normal and PCOS theca cells following 24 h treatment with and without 20 µM forskolin serum free medium. CYP11A1 mRNA was measured using quantitative real-time PCR as described in Materials and Methods. The data presented in **Panel A** and **Panel B** are data obtained from two normal and two PCOS patients, that are representative of data collected from theca cells from 5 normal and 5 PCOS patients. The data presented in **Panel C** are the results from independent analyses of theca cells isolated from 5 normal and 5 PCOS women. CYP11A1 mRNA accumulation was increased in PCOS theca cells as compared to normal theca cells under both control (*a*, *P*<0.01) and forskolin-stimulated conditions (*b*, *P*<0.01). Forskolin- treatment significantly increased CYP11A1 mRNA accumulation in both normal and PCOS theca cells (*, *P<0.01*).

For quantitative real-time PCR, total RNA was isolated [19] from theca cells that were grown to subconfluence, transferred into serum-free medium, containing DMEM/F12 1.0 mg/mL BSA, 100 µg/mL transferrin, 20 nM insulin, 20 nM selenium, 1.0 µM vitamin E and antibiotics, and treated as indicated. RNA (1 µg) samples were then reverse transcribed using oligo (dT) and 200 units Stratascript Reverse Transcriptase (Stratagene, Cedar Creek, TX). CYP11A1 mRNA abundance was determined by quantitative Real-time PCR as previously described [29], using a gene specific two-step PCR and carried out in triplicate for each cDNA sample as well as a series of serial dilutions in a Mx3000P Thermocycler (Stratagene, Cedar Creek, TX). For quantitation of CYP11A1 (Fig. 1), we utilized the forward primer (5′-GAGGGAGACGGGCACACA-3′), the reverse primer (5′-TGACATAAACCGACTCCACGTT-3′) and CYP11A1 specific probe (5′-TCCACCTTCACCATGTC CAGAAT-3′). 18S ribosomal and/or TATA-binding protein quantitated (TBP) mRNA abundance was utilized for data normalization as noted. For quantitation of TBP we utilized the forward primer (5′- F-CACGGCACTGATTTTCAGTTCT-3′), the reverse primer (5′-TCTTGCTGCCAGTCTGGACT-3′) and TBP specific probe (5′- TGTGCACAGGAGCCAAGAGTGAAGA - 3′). For quantitation of 18S we utilized primer and probe sets from Invitrogen. For determination of mRNA half-life, theca cells maintained in serum-free media for 24 h (time zero) were treated with 75 µM of the transcription inhibitor 5, 6-dichloro-benzimidazole 1-β-D-ribofuranoside (DRB) for 0 to 48 h. The rate of decay (*k*) was assessed by measuring the amount of CYP11A1 mRNA at each timepoint, over the amount at time zero, and determined by nonlinear regression (Prism 5.0, GraphPad Software, San Diego, CA). Half-life (t_1/2_) of transcripts/mRNA were calculated from the *k* based on the equation, t_1/2_ = (ln 2)/*k*.

### Construction of *CYP11A1* promoter constructs

**Figure 2:**
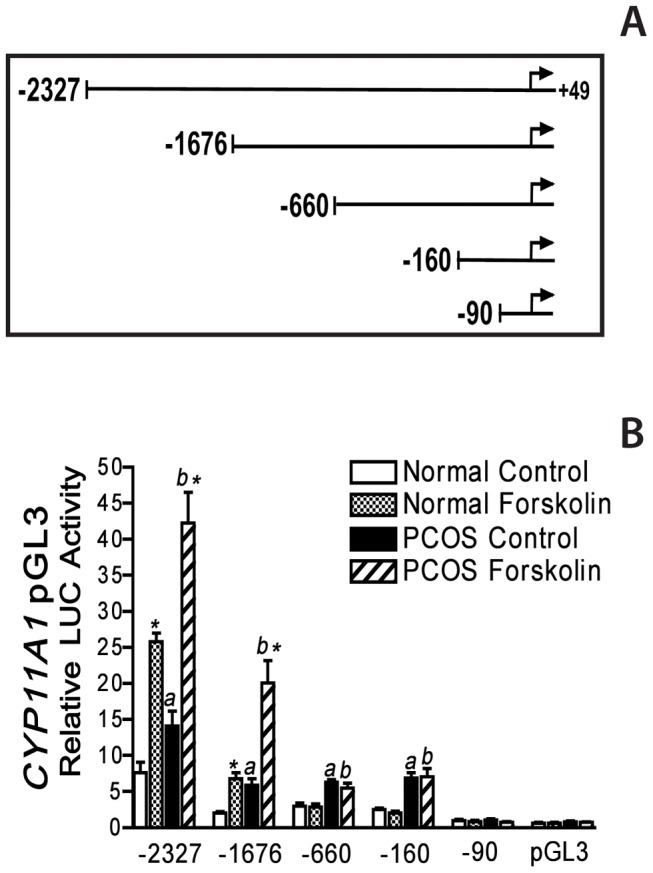
Deletion analysis of the *CYP11A1* promoter in normal and PCOS theca cells. **A)** Theca cells were transiently transfected with pGL3 luciferase constructs containing −2327, −1676, −660, −160, or −90 to +49 bp of the 5′-flanking sequence of the *CYP11A1* gene. All constructs contain the endogenous TATA box and transcriptional start site. **B)** Normal and PCOS theca cells were transiently transfected with the above constructs described in Materials and Methods. Following transfection, cells were cultured in transfection medium alone or with forskolin (20 µM) for 48 h. Data are presented as relative luciferase (LUC) activity that was normalized with β-galactosidase activity, and represent the mean ± SEM of independent experiments in five normal and five PCOS theca cell cultures. *CYP11A1* promoter activity was increased in PCOS theca cells, under basal (*a*, *P*<0.01) and forskolin-stimulated conditions (*b*, *P*<0.01), as compared to normal theca cells for individual promoter constructs.

**Figure 3:**
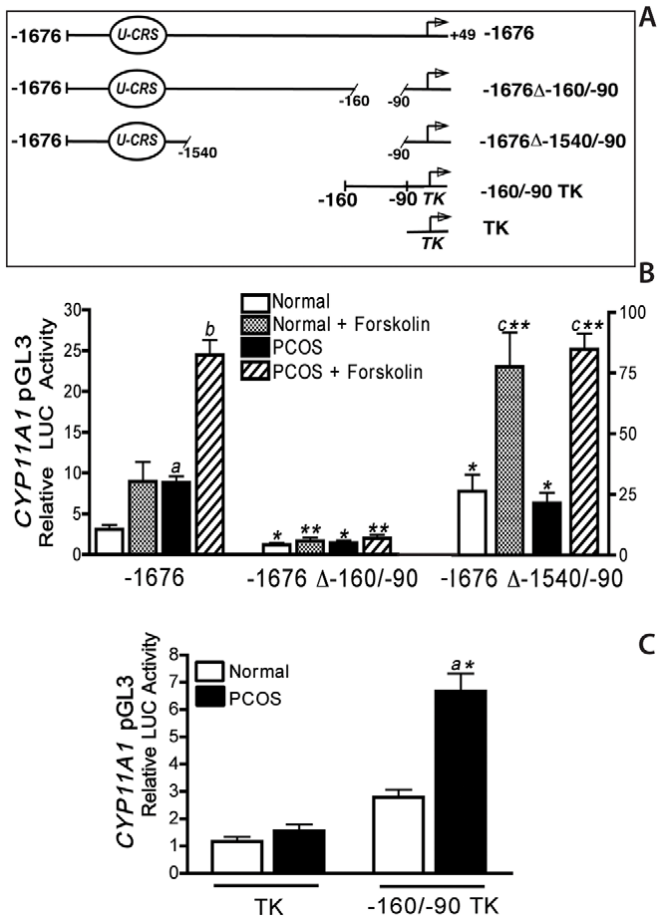
Differential regulation of the minimal −160/−90 bp *CYP11A1* promoter region in normal and PCOS theca cells. **A)** The full-length −1676 *CYP11A1* construct, the −1676 construct that lacks the −160/−90 bp region but retains sequences from −90 to +49 bp (−1676Δ−160/−90), a −1676 construct containing the sequence between −1676 to −1540 bp fused the minimal −90 *CYP11A1* promoter construct (−1676Δ−1540/−90) containing a putative *U-CRS* element, and constructs containing the thymidine kinase promoter alone (TK) or with the −160/−90 bp upstream of the TK promoter (−160/−90 TK). **B)** Normal and PCOS theca cells were transiently transfected with the−1676, −1676Δ−160/−90, −1676Δ−1540/−90 promoter constructs as described in Materials and Methods. Following transfection, cells were cultured in transfection medium alone or with forskolin (20 µM) for 48 h **C)** Normal and PCOS theca cells were transfected with promoter constructs containing either TK, or with −160/−90 TK. Following transfection, cells were cultured in transfection medium for 24 h. Data are presented as relative luciferase (LUC) activity that was normalized with β-galactosidase activity, and represent the mean ± SEM of independent experiments in four normal and four PCOS theca cell cultures. Both basal (*a*, P<0.01) and forskolin (*b*, P<0.01) stimulated −1676 *CYP11A1* promoter regulation is increased in PCOS theca cells. In normal theca cells, −1676Δ−1540/−90 *CYP11A1* activity was significantly increased under basal conditions (*, *P*<0.01), and forskolin-treatment (**, *P*<0.01) conditions, as compared to the full-length −*1676 CYP11A* construct. In contrast, in PCOS theca cells −1676Δ−1540/−90 *CYP11A1* activity was significantly increased under basal conditions (*, *P*<0.01), and forskolin stimulated (**, *P*<0.01) conditions, as compared to the full-length −*1676 CYP11A1* construct (Fig. 3B). These data demonstrate that 70 bp sequence between −160/−90 bp of the start site of transcription of the *CYP11A1* gene confers increased basal expression in PCOS theca cells. The U-CRS element between −1676 to 1540 of the promoter confers basal and cAMP-dependent regulation in both normal and PCOS theca cells.

The −2327 and −1676 *CYP11A1* LUC, containing −2327 or −1676/+49 bp of the 5′-flanking sequence of the human *CYP11A1* promoter in pGL3-basic (Fig. 2A), have been previously described [25], [33]. Sequentially smaller fragments of the human *CYP11A1* gene promoter were generated by PCR amplification using the following forward primers (−660 *CYP11A1*, 5′-cctgagctcCAGAGTGGAGCCTGACCA-3′; −160 *CYP11A1*, 5′-cctgagctcACGCTGCAGAAATTC CAG-3′; −90 *CYP11A1*, 5′-cctgagctcTGCAGCAGGAG GAAGGA -3′) and a reverse primer corresponding to the pGL3-basic polylinker (5′gccaagcttACTTAGATCGCA GATCTG-3′). PCR product was amplified by 20 cycles of Expand Long Template PCR (Roche Diagnostics, IN), and subcloned (Sac I/HindIII) into the pGL3 basic luciferase vector (Promega). The deletion construct −1676Δ (−160/−90) was generated by PCR amplification of a sequence from −1676 to −160, using the forward primers −1676 (5′-gccggtacCTCATCACCCTGCCGC TGC-3′) and a −160 reverse primer (5′- cctgagCTCTTCTCCAAAGGAC AGG-3′), followed by Kpn I and Sac I digestion and ligation into the −90 LUC construct (Fig. 3A). For the deletion construct −1676Δ(1540/−90) LUC, oligonucleotides corresponding to −1676/−1540 were annealed and ligated into the −90 LUC construct (Fig. 3A). For −160/−90 TK LUC, oligonucleotides corresponding to −160/−90 were annealed, digested with Sac I and Bgl II, and ligated into a thymidine kinase (TK)-pGL3basic vector. The TK-pGL3basic was constructed by subcloning the HSV TK promoter (BglII/HindIII) from pRL-TK (Promega, Madison, WI) into pGL3-basic (Fig. 3A). All constructs were confirmed by automated DNA sequencing.

### Construction of CYP11A1 UTR luciferase constructs

The 5′-UTR CYP11A1/LUC construct was generated by ligating annealed synthetic oligonucleotides corresponding to the 5′-UTR of CYP11A1 mRNA into NcoI/HindIII sites of the pGL3 control vector (Promega, Madison, WI) located upstream (5′) of the luciferase coding sequence. The construct was confirmed by automated DNA sequencing.

### Transient transfection of normal and PCOS theca cells

Human theca cells isolated from normal cycling women and women with PCOS were transfected as previously described [22], [30], [34] using the modified calcium phosphate method of Graham and Van der Eb [34]. Theca cells were grown in growth medium as described above. Sixteen hours prior to transfection, the cells were sub-cultured at a 1∶6 ratio in growth medium. One hour prior to transfection, the cells were transferred into transfection medium containing, DME high-glucose medium containing 20 mM HEPES and 2% heat-inactivated calf serum (Atlanta Biologicals, Atlanta, GA), and moved to a 3% CO_2_, 95% ambient air, 37°C incubator. The cells were then transfected with calcium phosphate precipitate containing 2 µg/dish of luciferase plasmid and 0.1 µg/dish of an expression vector for β-galactosidase, pSVβ-gal (Promega, Madison, WI) and 20 mM CaCl_2_, per 30 mm well, as indicated. To increase transfection efficiency, 6 h following transfection, the cells were glycerol shocked with a solution containing 15% glycerol in 25 mM Hepes buffer containing 140 mM NaCl and 0.5 mM Na_2_HPO_4_, and transferred into transfection media with and without 20 µM forskolin for 48 h. The cells were then were harvested with trypsin, pelleted, and resuspended in reporter lysis buffer (Promega). Luciferase activity was determined with the Luciferase Assay System (Promega) on a Sirius Luminometer (Zylux Corp., Oak Ridge, TN). β-Galactosidase activity was measured by the chemiluminescent assay Galacto-Light Plus (Tropix, Bedford, MA) and utilized for normalization of transfection efficiency.

### Cytoplasmic Extract Preparation

Human theca cells were transferred into DMEM/F12 serum-free medium containing 1.0 mg/mL BSA, 100 µg/mL transferrin, 20 nM insulin, 20 nM selenium, 1.0 µM vitamin E and antibiotics, in the absence or presence of 10 µM forskolin. At 24 h, cells were harvested with trypsin/EDTA and cytoplasmic extracts were prepared in buffer containing 0.1% NP-40, 20 mM HEPES (pH 7.9), 20 mM sodium chloride, 1 mM dithithreitol, 0.5 mM PMSF, 0.2 mM EDTA, 2 µg/mL leupeptin, 1 mM benzamidine, 1 mM sodium orthovanadate, and 20 mM sodium fluoride to inhibit protein phosphatases and proteases, as previously reported [23]. Protein concentrations of cytoplasmic extracts were determined by BCA protein assay [23].

### In vitro degradation of CYP11A1 mRNA

*In vitro* mRNA decay reactions were performed as previously described [23], [35]. Various lengths of the CYP11A1 cDNA were generated by PCR amplification of a pBS-CYP11A1 plasmid containing the full-length (1.86 kbp) CYP11A1 cDNA. The PCR products contained a T7 promoter site and were used to synthesize biotinylated CYP11A1 transcript in the MAXI script kit (Ambion, Austin, TX). Reactions included 0.3 µg of cytoplasmic extract and approximately 10 ng of biotinylated transcript in RNA degradation buffer comprised of 0.6 U recombinant RNAsin (Promega). Components were combined on ice, mixed, and incubated at 37°C. For comparison of decay rates for transcripts of different length, equamolar amounts of biotinylated RNA were included in the reactions. At each timepoint from 0–60 min, 10 µl of the reaction was removed and immediately precipitated in cold 70% ethanol containing Glycoblue co-precipitant (Ambion, Austin, TX). Following resuspension, the reactions were separated on a 1.5% agarose/formaldehyde gel and transferred to nylon membrane. Biotinylated RNA was detected using the Bright Star BioDetect kit (Ambion, Austin, TX) and quantitated using a GeneGnome bioimager and GeneSnap v6.03 and GeneTools v3.03 (Syngene Bioimaging, Cambridge, UK)

### Statistical Analysis

Data are presented and described in the text as the mean ± SEM from transfections or mRNA decay analysis (t_1/2_) performed in triplicate in 5 independent normal and 5 independent PCOS theca cells cultures unless otherwise noted. The results from QRT-PCR, mRNA decay analysis (t_1/2_), and transfection analysis were collected from individual patients and one-way *ANOVA* was performed using Prism 3.0c (GraphPad Software, San Diego, CA). *P* values were determined by the Tukey method for multiple comparisons when significant differences were indicated by one-way ANOVA.

## Results

### Comparison of steady state of CYP11A1 mRNA abundance in theca cells from normal cycling and PCOS women

Quantitative real-time PCR was utilized to examine the time course of CYP11A1 mRNA in theca cells isolated from normal and PCOS patients. Cells were grown until subconfluent in growth medium then transferred into serum-free medium, and treated for 4, 8, 16, 24, or 48 h in the presence and absence of 20 µM forskolin. At time zero prior to treatment, and the end of each time point, the plates of cells were flash frozen, cells were harvested, RNA was prepared, and CYP11A1 mRNA abundance was quantified as described in Materials and Methods. As shown in Figs. 1A–B, CYP11A1 mRNA can be measured at time zero in both normal and PCOS theca cells and appears to be increased in PCOS theca cells in the absence of treatment. As shown in Fig. 1B, increased steady state CYP11A1 mRNA can be observed at 4–8 h in normal and PCOS theca cells, under both basal and forskolin-stimulated conditions. Under basal conditions, the amount of CYP11A1 mRNA accumulation was similar following 8–48 h in serum-free medium. Maximal induction of CYP11A1 mRNA was achieved following 24 h treatment with forskolin in both normal and PCOS theca cells (Figs. 1A–1B).

To compare steady state levels of CYP11A1 mRNA in normal and PCOS theca cells, CYP11A1 mRNA abundance was examined in normal and PCOS theca cells that had been grown until subconfluent and transferred into serum-free medium with and without 20 µM forskolin for 24 h. Following treatment, total mRNA was harvested and CYP11A1 mRNA was quantitated using real-time PCR as described in Materials and Methods. As shown in Fig. 1B, CYP11A1 mRNA abundance is ∼2–2.5-fold higher in PCOS theca cells as compared to normal cells, under both basal (*a*, *P*<0.01) and forskolin-stimulated (*b*, *P*<0.01) conditions (Fig. 1B). Forskolin- treatment significantly increased CYP11A1 mRNA accumulation in both normal and PCOS theca cells (*, *P<0.01*). These data are in agreement with our previously published Northern analysis and qRT-PCR indicating that PCOS theca cells contain elevated steady state CYP11A1 mRNA levels [19], [25].

### Deletion analysis of the *CYP11A* promoter

Previous examination of the human CYP11A1 promoter function in theca cells indicated that sequences within −1676 bp of the start site of transcription conferred transcriptional activity and increased promoter function in PCOS [25]. To examine the regions involved in increased promoter regulation in PCOS theca cells, a series of promoter constructs containing successive deletions of the 5′-flanking sequence of the human *CYP11A1* gene were generated. Luciferase constructs containing −2327, −1676, −660, −160, or −90 to +49 bp of the *CYP11A1* promoter were transiently transfected into theca cells isolated from normal cycling women and women with PCOS (Fig. 2A). In order to examine the regions of the *CYP11A1* promoter involved in basal, as well as cAMP-dependent regulation, the cells were cultured in the absence (basal) or presence of 20 µM forskolin for 48 h.

Comparison of *CYP11A*1 promoter function in normal and PCOS theca cells showed that under both basal (*a*, *P*<0.01) and forskolin-stimulated (*b*, P<0.01) conditions, −2327, −1676, −660, and −160 *CYP1A1* LUC activity was increased approximately 2-fold in PCOS theca cells, as compared to normal theca cells (Fig. 2B). In contrast, −90 *CYP11A1* promoter function was not different in normal and PCOS. A significant reduction in basal and cAMP- dependent *CYP11A1* promoter function was observed following exclusion of sequences upstream of −1676 bp, and −660 bp of the start site of transcriptions as well as −90 bp, in both normal and PCOS cells. These data suggest the presence of regulatory regions within −2327 to −660 bp, as well as −160 to −90 bp that contribute *CYP11A1* promoter regulation in normal and PCOS theca cells.

Treatment with forskolin resulted in significant increases in promoter function for both the −2327 and −1676 *CYP11A1* constructs (*, *P<0.01*). Whereas, deletion of sequence 5′ of −660 bp significantly reduced forskolin-induction of *CYP11A* promoter constructs, indicating that sequences between −1676 and −660 bp are required for cAMP-dependent regulation in both normal and PCOS theca cells (Fig. 2B). The robust increase in basal and cAMP-dependent reporter activity observed in following transfection of the −2327 bp and −1676 bp constructs, most likely is the result of regulation through a previously described U-CRS response elements, TCAAGGTCA located between −1640 to −1553 bp, and −1931 to −1822 of the *CYP11A* promoter which has SF-1 and CRE like binding sites, and thus confers basal and cAMP-dependent regulation. Removal of sequences upstream of −660 bp resulted in a similar 2- to 3- fold reduction in basal and forskolin-stimulated *CYP11A1* promoter function in both normal and PCOS cells.

−660 and −160 *CYP11A1* promoter function was increased 2-fold in PCOS theca cells as compared in normal cells (*a*, *P*<0.01). Both of these shorter *CYP11A1* promoter constructs lacked cAMP responsiveness. Deletion of sequences between −160 to −90 bp, reduced basal promoter function and ablated *CYP11A1* promoter function in both cell types. Together, these data suggest that increased *CYP11A1* promoter function in PCOS theca cells results primarily from augmented basal regulation of an element within the general boundaries of −160 bp to −90 bp of the start site of transcription of the *CYP11A1* gene (Fig. 2B).

### Increased CYP11A1 promoter regulation in PCOS theca cells

To determine whether the sequence between −160 to −90 bp of CYP11A1 promoter are necessary for increased basal regulation in PCOS theca cells, we constructed −1676 *CYP11A1* promoter constructs in which sequences between −160 to −90 bp (−1676 Δ−160/−90) were deleted (Fig. 3A). In addition, to determine the extent to which the putative *U-CRS* consensus element confers both basal and cAMP dependent regulation in normal and PCOS theca cells, we constructed a *CYP11A1* reporter construct containing the sequence between −1676 to −1540 bp fused to the minimal −90 *CYP11A1* promoter construct (−1676Δ−1540/−90). Both of these constructs were compared to the full-length −1676 *CYP11A1* promoter construct following transient transfection analysis under basal and forskolin (20 µM) stimulated conditions (Fig. 3B).

Compared to the full-length −1676 *CYP11A1* promoter, the function of −1676Δ−160/−90 was significantly reduced in both normal and PCOS theca cells under both basal (*, *P*<0.01) and forskolin-stimulated conditions (**, *P*<0.01). In contrast to the −1676 promoter, whose function is >2-fold higher in PCOS theca cells, there was no difference in −1676 Δ−160/−90 promoter function in normal and PCOS cells (Fig. 3B). Given that the deletion of −160/−90 bp reduces promoter function in both normal and PCOS theca cells, and that lower promoter function was also observed in the serial deletion of sequences from −160 to −90, it is likely that basal regulatory sequences are found within this region that confer increased regulation in PCOS theca cells.

Transient transfection analysis of the −1676Δ−1540/−90 *CYP11A1* construct in normal and PCOS theca cells resulted in almost identical increases in basal and forskolin-stimulated luciferase activity (Fig. 3B), which were significantly higher than that observed with the full length −1676 *CYP11A1* construct. In both normal and PCOS theca cells, forskolin stimulated −1676Δ−1540/−90 *CYP11A1* reporter activity increased over two-fold (c, *P*<0.01) compared to basal, non-stimulated values. These data confirm those in the literature. demonstrating that the *U-CRS* element between −1640 to −1553 bp of the promoter confers basal and cAMP-dependent regulation in both normal and PCOS theca cells. In normal theca cells, −1676Δ−1540/−90 *CYP11A1* activity was significantly increased under basal conditions >5-fold (*, *P*<0.01), and >7-fold following forskolin-treatment (**, *P*<0.01) conditions, as compared to the full-length −*1676 CYP11A* construct (Fig. 3B). In contrast, in PCOS theca cells −1676Δ−1540/−90 *CYP11A1* activity was significantly increased under basal conditions >2-fold (*, *P*<0.01) and >3-fold following forskolin-treatment (**, *P*<0.01) conditions, as compared to the full-length −*1676 CYP11A* construct (Fig. 3B). These data suggest that sequences between −1540 and −90 bp of the *CYP11A1* promoter may confer differential regulation in normal and PCOS theca cells.

To determine if the −160/−90 region of the *CYP11A1* promoter confers increased activity in PCOS theca cells, we transfected normal and PCOS theca cells with luciferase reporter constructs containing the −160/−90 bp region fused to a heterologous thymidine kinase promoter (Fig. 3A) or a luciferase construct containing the empty TK promoter (TK). As shown in Figure 3C, transfection of the empty luciferase construct, TK, resulted in low but measureable levels of luciferase activity in normal and PCOS theca cells, which were not significantly different. Transfection of normal theca cells with the −160/−90 bp region of the *CYP11A1* promoter fused upstream of the minimal thymidine kinase promoter (−160/−90 TK) resulted in a ∼2-fold increase in luciferase activity as compared to the empty TK construct, which was not statistically significant. Transfection of the −160/−90 TK construct in PCOS cells resulted in a >4-fold increase compared to the empty TK construct (*a*, *P*<0.01), and was increased 2-fold as compared to normal theca cells (*, *P*<0.01). These data demonstrate that sequences within −160 to −90 bp of the *CYP11A* promoter are necessary for increased *CYP11A1* promoter function in PCOS theca cells and contribute to increased basal regulation.

### NF-1C2 regulation of the *CYP11A1* promoter

In studies examining the basis for increased CYP17 gene expression in normal and PCOS theca cells, we previously reported that a16 bp element between −180 tp −144 bp of the CYP17 promoter that confers increased basal regulation in PCOS theca cells. We demonstrated that transcription factor NF-1C2, had the capacity to bind to this 16 bp minimal element and inhibit (*i.e.*, repress) the *CYP17A1* promoter. Moreover, we reported that NF-1C2 protein levels were reduced in PCOS theca cells, suggesting that a decrease in NF-1 repression may be involved in increased *CYP17A1* gene expression in PCOS theca cells.

**Figure 4:**
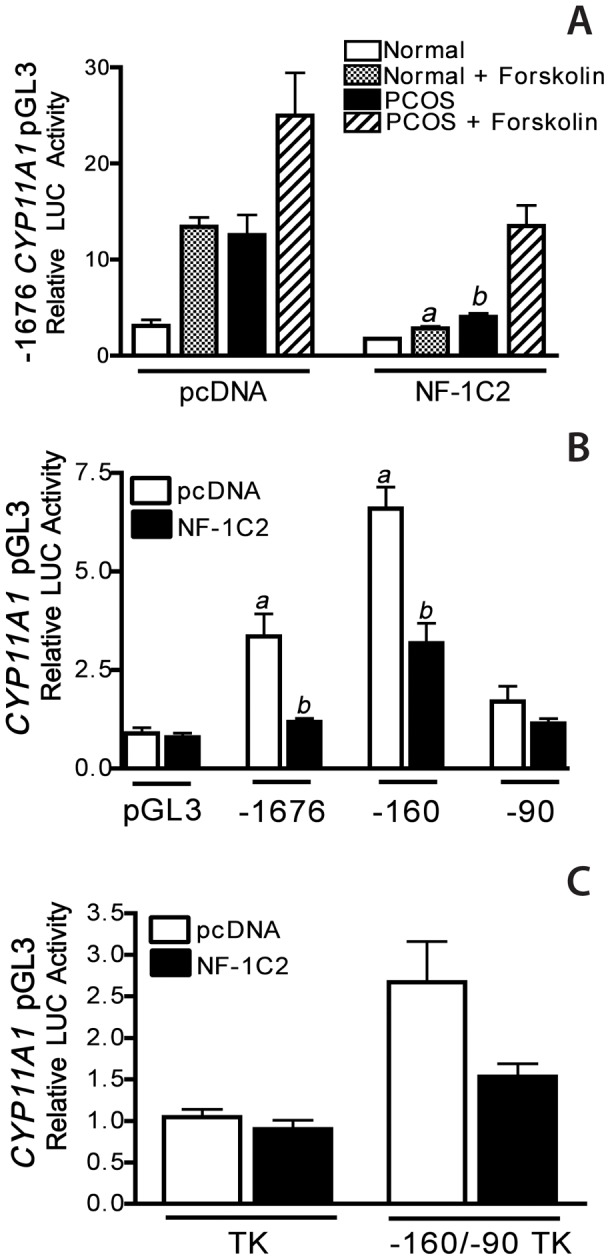
NF-1C2 regulation of the *CYP11A1* promoter in theca cells. **A)** The effect of human NF-1C2 on *CYP11A1* promoter function was examined in normal and PCOS theca cells transiently transfected with the −1676 *CYP11A1* luciferase construct and co-transfected with an empty pcDNA plasmid, or a pcDNA plasmid expressing NF-1C2. Following transfection, cells were cultured in transfection medium with and without 20 µM forskolin (F) for 48 h. To determine the sequences in the *CYP11A1* promoter that confer NF-1C2 regulation, cells were transfected with a **B**) pGL3 constructs containing −1676, −160, or −90 to +49 bp of the 5′-flanking sequence of the CYP11A1 gene, or **C)** the minimal −160/−90 TK and empty TK constructs, following co-transfection with pcDNA plasmid expressing NF-1C2 or an empty pcDNA plasmid, the cells were cultured in serum free medium for 48 h. All data are presented as relative luciferase (LUC) activity that was normalized with β-galactosidase activity and represent the mean ± SEM of multiple independent experiments. These experiments demonstrate that NF-1C2 inhibits both basal (*a*, *P*<0.01) and forskolin (*b*, *P*<0.01) stimulated −1676 *CYP11A1* promoter function in normal and PCOS theca cells, as well as basal −160 *CYP11A1* promoter function (*a*, *P*<0.01) in PCOS theca cells. Moreover, sequences between −160/−90 bp of the *CYP11A1* promoter confer NF-1C2 inhibition.

Examination of the −160/−90 bp *CYP11A1* minimal element suggests sequence similarity with the bipartite recognition sequence ((C/T)TGGC(N)_6_CC(N)_3_) for NF-1 [36], [37]. To examine the possibility that NF-1C2 coordinately regulates CYP11A1 gene expression in a manner similar to the *CYP17A1* promoter, we performed studies to examine the effect of a human NF-1C2 pcDNA plasmid or an empty pcDNA plasmid on the full length −1676 *CYP11A1* promoter construct in normal and PCOS theca cells. Following transfection, the cells were treated with and without 20 µM forskolin. As shown as a comparison in Fig. 4A, co-transfection with empty pcDNA plasmid alone had no effect on −1676 *CYP11A1*/LUC promoter function, and both basal and forskolin-stimulated reporter function remained significantly augmented in PCOS theca cells, as compared to normal theca cells (*a, b*, *P*<0.01). In contrast to empty pcDNA plasmid, co-transfection with NF-1C2 plasmid markedly inhibited basal and forskolin-stimulated −1676 *CYP11A1* promoter function in normal and PCOS theca cells. In PCOS theca cells, basal promoter function was significantly inhibited (*b*, *P*<0.01), and NF-1C2 co-transfection of PCOS cells, inhibited *CYP11A1* promoter activity (Fig. 4A). These data show that overexpression NF-1C in PCOS theca cells has the ability to decrease *CYP11A1* transcription, and suggest that the previously reported increase in NF-1C levels observed in normal theca cells contribute to lowered levels of *CYP11A1* expression which are phenotypic of the normal cycling ovary.

To identify sequences of the *CYP11A1* promoter that confer NF-1C2 regulation, theca cells were transfected with pGL3 constructs containing −1676, −160, or −90 to +49 bp of the 5′-flanking sequence of the *CYP11A1* gene with the empty pcDNA plasmid or NF-1C2 plasmid. In these experiments, we examined differences in basal expression in the absence of forskolin, because basal *CYP17A1* and *CYP11A1* promoter regulation are both conferred by basal elements in PCOS theca cells. As shown in Fig. 4B, co-transfection with pcDNA, or NF-1C2 has no effect on pGL3 or −90/LUC activity in PCOS theca cells. Both −1676 and −160 *CYP11A1*/LUC promoter function are increased in theca cells following pcDNA co-transfection (a, *P*<0.01), and NF-1C2 significantly (b, *P*<0.01) inhibits both of these activities in excess of 50–75% (Fig. 4B), further suggesting that sequences between −160 to −90 of the start site of transcription of the *CYP11A1* promoter may confer NF-1C2 repression. To test this possibility, we transfected PCOS theca cells with the *CYP11A1* promoter construct containing the −160 to −90 sequence, −160/−90 TK (see Fig. 4C) and the empty control TK constructs with the pcDNA plasmid expressing NF-1C2 or the empty pcDNA plasmid. Following transfection the cells were cultured in serum free medium for 24 h. These experiments demonstrate that NF-1C2 inhibits both basal and forskolin stimulated *CYP11A1* promoter function in normal and PCOS theca cells. Moreover, sequences between −160/−90 bp of the *CYP11A1* promoter confer NF-1C2 inhibition.

### Endogenous CYP11A1 mRNA stability

**Figure 5:**
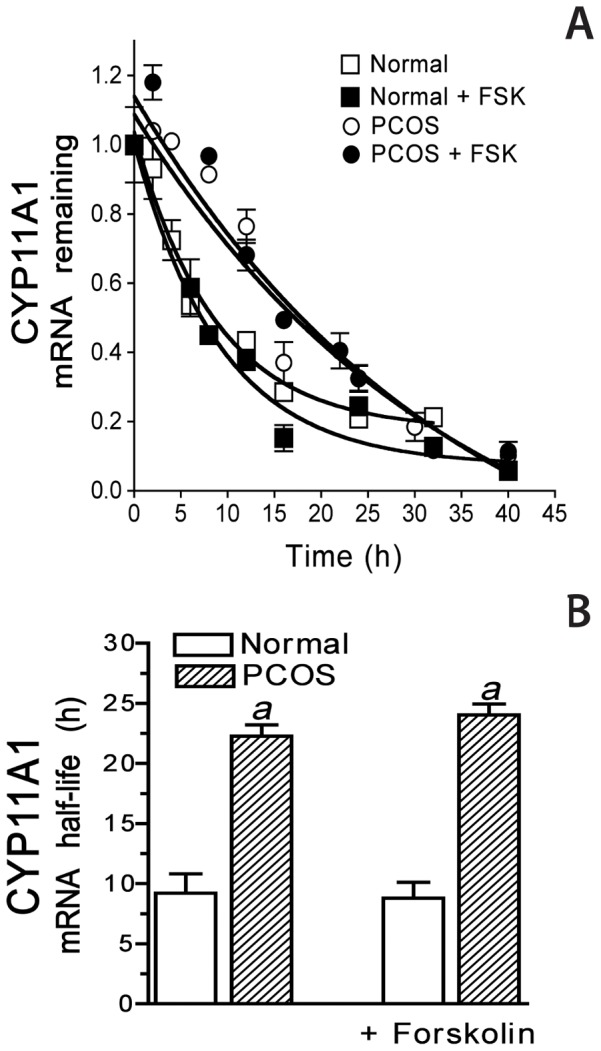
Endogenous CYP11A1 mRNA half-life in normal and PCOS theca cells. The stability of endogenous CYP11A1 mRNA was examined in normal and PCOS theca cells under untreated and forskolin (20 µM)-stimulated conditions following treatment with the transcriptional inhibitor DRB (75 µM) from 2–42 h. A) Graphical representation of the amount of CYP11A1 mRNA remaining at each time interval, determined by quantitative real-time PCR. **B)** The half-life of endogenous CYP11A1 mRNA is presented as the mean ± SEM from independent examinations in 5 normal and 5 PCOS theca cell cultures. CYP11A1 mRNA half-life was increased in PCOS theca, under both untreated (*a*, *P*<0.01) and forskolin-stimulated conditions (*a*, *P*<0.01). Forskolin treatment did not significantly alter CYP11A1 mRNA half-life in normal or PCOS theca cells.

Given our previously published observations of differences in mRNA stability of CYP17A1 and GATA6 mRNAs in PCOS theca cells [23], [38], we also determined the stability (half-life) of CYP11A1 mRNA in normal and PCOS theca cells. For these studies, the decay of endogenous CYP11A1 mRNA over time was examined under conditions where transcription was pharmacologically blocked using the transcriptional inhibitor 5, 6-dichloro-benzimidazole 1-ß-D-ribofuranoside (DRB) [23], [39]. To induce CYP11A1 mRNA, normal and PCOS theca cells were incubated in serum-free media for 24 h, either in the absence or presence of forskolin, prior to addition of 75 µM DRB. CYP11A1 mRNA abundance was then measured by QRT-PCR analysis at various time points (Fig. 5A) and the fraction of transcript remaining was utilized to calculate the half-life of the CYP11A1 mRNA as described in Materials and Methods. Half-life values for CYP11A1 mRNA were determined from 4 independent normal and 4 independent PCOS theca cell cultures and are presented in Figure 5B. For normal theca cells, the decay of CYP11A1 mRNA occurred with a half-life of 9.22±1.62 h under basal conditions, and 8.80±1.33 h after forskolin treatment. In PCOS theca cells, CYP11A1 mRNA decay occurred at a slower rate with a half-life of 22.28±0.95 h under basal conditions, and 24.05±0.92 h in the presence of forskolin treatment. The half-life of CYP11A1 mRNA was extended by approximately 13–17 h in PCOS theca cells (*a*, *P*<0.01) under both basal and forskolin-stimulated conditions. Forskolin treatment, activation of adenylate cyclase, and increased cAMP, did not significantly affect CYP11A1 mRNA half-life in either normal or PCOS cells.

### In vitro CYP11A1 mRNA decay and examination of the 5′-UTR of CYP11A1 mRNA

**Figure 6:**
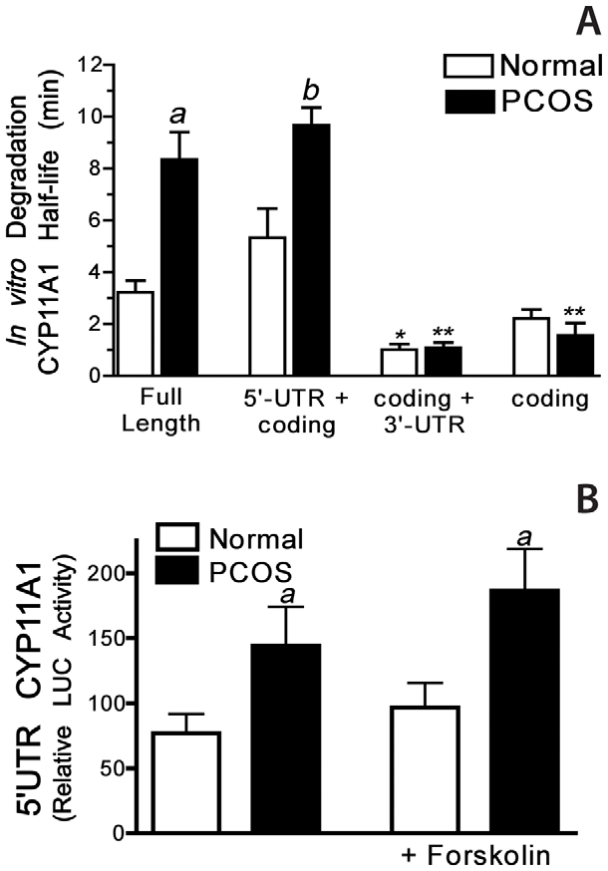
The 5′UTR of CYP11A1 mRNA confers increased stability in PCOS theca cells under basal conditions. **A)** The individual half-lives of various CYP11A1 RNA probes were determined using RNA *in vitro* degradation assays. Biotinylated CYP11A1 mRNA transcripts (i.e., the full-length transcript, 5′UTR+coding region, coding region alone, and coding region +3′UTR) were incubated with cytoplasmic extracts isolated from either normal or PCOS theca cells. The stability of each transcript over time (half-life) is presented as the mean ± SEM of five independent assays. The results of these experiments demonstrated that the stability of CYP11A1 RNA transcripts containing either the full-length (*a, P*<0.01) or 5′+coding region (*b, P*<0.01), were significantly increased in assays using PCOS extracts, compared with normal extracts. The coding transcript+3′-UTR was markedly reduced in normal (*, *P<0.01*) and PCOS (**, *P<0.01*) theca cells as compared to the 5′UTR+coding transcript, and were similar in normal and PCOS cells. **B)** To examine functional differences in 5′UTR of CYP11A1 in normal and PCOS theca cells, both cell types were transiently transfected with a luciferase (LUC) construct containing the 5′ UTR of CYP11A1 mRNA and incubated in the absence (untreated) or presence of forskolin (20 µM) for 48 h. Data are presented as relative luciferase (LUC) activity following normalization by ß-galactosidase and represent the mean ± SEM from transfections performed in triplicate in 4 independent normal and 4 independent PCOS theca cells cultures. Luciferase expression of the 5′ UTR construct was significantly higher in PCOS theca cells as compared to normal cells (*a, P*<0.05). Forskolin treatment had no effect on CYP11A1 stability in normal or PCOS theca cells.

To further examine differences in CYP11A1 mRNA stability in normal and PCOS theca cells, *in vitro* degradation assays were performed as previously described in our laboratory [23]. In order to determine the region(s) of the CYP11A1 mRNA involved in differential regulation of mRNA stability, *in vitro* degradation assays were performed utilizing biotinylated CYP11A1 RNA transcripts corresponding to the full length transcript, the coding region alone, the 5′-UTR+coding region, or the 3′-UTR+coding region (Fig. 6A). In these assays the biotinylated RNA transcripts were incubated with cytoplasmic extracts prepared from normal and PCOS theca cells that were grown until subconfluent then placed in serum free medium for 24 h, as described in Materials and Methods. *In vitro* half-lives of synthesized transcripts are substantially shorter than endogenous half-lives, however, *in vitro* degradation assays indicate rank orders of decay which reflects relative differences in message stability among samples. As shown, the half-life of the full-length CYP11A1 transcript was increased >2-fold in PCOS extracts (*a*, *P<0.01*), as compared to normal extracts (Fig. 5B). The half-life of the 5′-UTR+coding transcript was approximately 2-fold longer in PCOS extracts (*b*, *P<0.01*) as compared to normal extracts (Fig. 6A). In contrast, the coding transcript+3′-UTR was markedly reduced in normal (*, *P<0.01*) and PCOS (**, *P<0.01*) theca cells as compared to the 5′UTR+coding transcript, and were similar in normal and PCOS cells. The stability of the coding region alone was significantly reduced as compared to full-length transcript and the 5′UTR+coding transcript in PCOS cells (**, *P<0.01*), and was not different in normal and PCOS. The stability of the β-actin transcript was similar in normal and PCOS extracts [23]. These data suggest that the 5′-UTR alone, rather than the 3′UTR or coding regions of CYP11A1 mRNA contributes to increased stability in PCOS. Furthermore, the 5′-UTR is required for the differential CYP11A1 mRNA stability observed in PCOS theca cells.

To examine the extent to which the 5′-UTR of CYP11A1 mRNA confers functional differences in reporter function in PCOS theca cells, we transfected normal and PCOS theca cells with a luciferase reporter construct containing the 5′-UTR of CYP11A1. As a control, replicate cultures of normal and PCOS cells were transfected with a control pGL3 luciferase construct. Following transfection the cells were treated with and without 20 µM forskolin for 48 hours. Experiments were performed in triplicate in 4 different normal and PCOS patient's cells. As shown in Figure 6B, 5′-UTR CYP11A1/LUC activity increased 2-fold in PCOS theca cells (*a*, *P*<0.01) as compared to normal theca cells, under both control and forskolin-stimulated conditions. Control pGL3 LUC activity was not different in normal and PCOS theca cells. These data suggest that the 5′UTR of CYP11A1 mRNA contributes to overall increased basal *CYP11A1* gene expression in PCOS theca cells.

## Discussion

In this report long-term cultures of theca cells isolated and propagated from normal cycling and PCOS women were utilized to compare the regulation of *CYP11A1* gene expression at the transcriptional and post-transcriptional levels. CYP11A1 mRNA accumulation was observed to be significantly increased, 3–4-fold, in PCOS theca cells as compared to normal theca cells maintained under basal or forskolin stimulated conditions (Fig. 1). Hence, in PCOS theca cells, there is an underlying increase in basal, steady state CYP11A1 mRNA accumulation, as compared to normal theca cells, suggesting that basal *CYP11A1* gene expression is augmented in PCOS theca cells.

These studies represent the first examination of the promoter elements involved in the regulation of CYP11A1 transcription in human theca cells. Transient transfection analysis of theca cells with reporter gene constructs containing successive serial deletions of the 5′ flanking region of *CYP11A1* promoter demonstrated that *CYP11A1* promoter regulation was differentially regulated in normal and PCOS cells (Fig. 2). More specifically, we observed that CYP11A1 promoter function was increased PCOS theca cells. Finer analysis of these deletion mutants in normal and PCOS theca cells, demonstrated that a region of the *CYP11A1* promoter between −1676 to −660 bp of the start site of transcription conferred a 2–3 fold increase in cAMP-responsiveness (*p*<0.01) in both normal and PCOS theca cells (Fig. 2). A minimal element, between −160 to −90 of the start site of transcription, conferred increased basal regulation in PCOS theca cells when compared to normal theca cells. In contrast, constructs containing −90 to +45 bp of the *CYP11A1* promoter, showed no basal or cAMP-dependent regulation, and were not observed to be different in normal and PCOS cells, or significantly different from transfection of a control pGL3 plasmid (Fig. 2).

Data demonstrating that serial deletion of sequences 5′ of −1676 and −660 bp of the CYP11A1 promoter ablated cAMP-responsiveness further suggested that sequences in these regions functionally acted like putative upstream cAMP (*U-CRS*) responsive elements in theca cells (Fig. 2). These studies show strong similarity to those examining cyclic AMP dependent regulation of *CYP11A1* transcription, and the *U-CRS* identified in adrenal Y1, placental JEG and human granulosa cells [40]. The CYP11A1 U-CRS cAMP-consensus element has been previously reported to consist of a core SF-1 binding site (U element, TCAAGGTCA), at −1617/−1609, and two flanking AP1/CBP sites (TGACTGAT), at −1666/−1626, and −1559/−1553 [16], [41], [42]. Transient transfection analysis of a the −1676Δ−1540/−90 *CYP11A1* construct was performed to examine whether the U-CRS conferred increased basal or cAMP regulation in normal and PCOS theca cells (Fig. 3A–3B). Results of these studies showed that the U-CRS, increased cAMP-dependent CYP11A1 to the same extent in normal and PCOS theca cells, but did not confer increased regulation in PCOS theca cells. These data confirm those in the literature demonstrating that the *U-CRS* element between −1640 to −1553 bp of the promoter confers basal and cAMP-dependent regulation in both normal and PCOS theca cells. In addition, demonstrating increased −1676Δ−1540/−90 promoter regulation compared to the full length −1676 CYP11A1 promoter construct, further provide evidence to suggest that sequences between −1540 and −90 bp of the *CYP11A1* promoter confer differential regulation in normal and PCOS theca cells (Fig. 3B).

In PCOS theca cells, significantly increased basal *CYP11A1* promoter regulation was conferred by a 70 bp proximal regulatory element located between −160 and −90 bp of the transcriptional start site. Examination of this nucleotide sequence revealed putative consensus binding sites for several transcription factors, including C/EBP-B, SF-1, COUP-TF, Trep-132, Sp-1, and NF-1. Specific examination of the −160/−90 bp *CYP11A1* minimal element revealed sequence similarity to the bipartite recognition sequence ((C/T)TGGC(N)_6_CC(N)_3_) for NF-1 [36], [37]. NF-1C, also referred to as CAAT-box transcription factor (CTF) [43], was the first NF-1 family member identified and has been reported to trans-activate and repress the transcription of a wide variety of genes expressed in developmental and tissue-specific patterns [44]–[47]. In human adrenal H295 cells, NF-1C was shown to bind the *CYP17A1* promoter, however functional studies were not reported [24]. We recently reported that the NF-1 family member NF-1C2, had the capacity to bind to and inhibit (i.e., repress) the *CYP17A1* promoter in PCOS theca cells. Moreover, our studies revealed that NF-1C2 protein levels in whole cell and nuclear extracts were reduced in PCOS theca cells. Combined, these data demonstrated that a reduction NF-1C2-dependent repression contributes to increased *CYP17A1* promoter activity and gene expression in PCOS theca cells, thereby increasing androgen biosynthesis. In view of the bipartite NF-1 consensus sequence in the minimal −160/−90 *CYP11A1* promoter, and sequence similarities between the minimal elements in the *CYP17* and *CYP11A1* promoters that conferred increased basal regulation in PCOS cells, studies were performed to investigate whether NF-1C2 coordinately regulates (*i.e.*, represses) the *CYP11A1* minimal promoter. Results of experiments examining the effects of co-transfection of NF-1C2 on a variety of *CYP11A1* promoter constructs (Fig. 6A–6C), showed that NF-1C2 had the capacity to inhibit luciferase activity of the full length −1676/+45, or −160/+45 and −160/−90TK *CYP11A1* promoter constructs. These data revealed that NF-1C2 had the capacity to repress *CYP11A1* promoter function through the −160/−90 bp element. In fact, deletion of the −160/−90 element that confers NF-1C2 regulation from the full length −1676 promoter construct, and is sufficient to convert the augmented *CYP11A1* promoter function observed in PCOS theca cells to levels observed in normal theca cells (Fig. 3B). Hence, given that NF-1C2 levels are reduced in PCOS theca cells, these data suggest that a reduction in NF-1C2 repression results in increased *CYP11A1* promoter expression in PCOS theca cells. Furthermore, they provide the first evidence to establish that NF-1C2 coordinately regulates both *CYP11A1* and *CYP17* gene expression in the PCOS ovary.

Numerous studies have established that the transcriptional regulation of *CYP11A1* gene expression involves specific transcription factors and co-factors that bind and interact with the *CYP11A1* promoter is tissue- and species- specific manner. However, to our knowledge there have been no studies that have examined the post-transcriptional regulation of *CYP11A1* gene expression at the level of mRNA stability in any steroidogenic tissue [41]. Gene expression profiles obtained from both steady state and newly transcribed mRNA (measured in nuclear run on studies) have revealed that the regulation of mRNA stability may account for as much as 50% of all measurements of changes in total cellular mRNA. Therefore, changes in the regulation of mRNA stability in a disease state can have significant implications on the up- or down- regulation of gene expression [48]–[50]. Regulation of mRNA turnover and stability is a major mechanism for controlling gene expression involving the interaction of cytoplasmic proteins, and in some cases microRNAs, that bind to regulatory regions on the 5′UTR, 3′ UTR, and/or coding regions of the mRNA [51]. In studies comparing normal and PCOS theca cells, determination of endogenous CYP11A1 mRNA half-life by pharmacological inhibition of transcription demonstrated that the half-life of CYP11A1 mRNA is increased two-fold in PCOS theca cells, as compared to normal theca cells. *In vitro* mRNA degradation studies and transient transfection of 5′UTR CYP11A1 luciferase reporter constructs demonstrated that the 5′-UTR confers increased stability to CYP11A1 mRNA in PCOS theca cells also approximately 2-fold under basal conditions. These studies indicate that a slower rate of CYP11A1 mRNA decay contributes to increased steady state basal mRNA accumulation and augmented *CYP11A1* gene expression in PCOS theca cells. In previous studies we also reported that CYP17A1 mRNA stability was increased in PCOS theca cells, under basal and cAMP-dependent stimulation [23]. We also demonstrated a similar requirement for the 5′-UTR of CYP17A1, with the added regulation of this 5′-UTR by forskolin stimulation. The factors that interact with the CYP17A1 or CYP11A1 mRNA are unknown, and RNA binding site analyses of both 5′-UTRs does not suggest common binding by known RNA binding factors. It is surprising that the 5′-UTRs of both of these key steroidogenic genes that are upregulated in PCOS are controlled by their 5′-UTRs. The majority of proteins that have been shown to regulate mRNA stability bind to 3′-UTRs. It is possible that the 5′UTR of CYP11A1 lacks cAMP-dependent regulation because it is only 44 bp, and is much smaller compared to the 212 bp 5′-UTR of CYP17A1. The regulation of steroidogenic enzyme expression by modifications in RNA stability provides an added complexity and level of regulation that will require further investigation to determine the key factors and signaling pathways involved in these processes. On the other hand, it is important to recognize that the 44 bp 5′-UTR confers a 2-fold increase in mRNA stability in PCOS theca cells.

Compared to their normal counterparts, PCOS theca cells in long-term culture produce increased amounts of steroids including progestins, androgen precursors such as DHEA, and testosterone. *CYP11A1* gene expression, the rate-limiting step in steroid biosynthesis is augmented in PCOS theca cells. This up-regulation of steady state *CYP11A1* mRNA accumulation in PCOS theca cells is associated with significant increases in both promoter activity and transactivation of the *CYP11A1* gene, and mRNA stability through the 5′-UTR of CYP11A1 mRNA.

In PCOS theca cells there is a coordinated increase in the expression of several steroidogenic enzymes, including *CYP17A1*, *3ß-HSDII*, Aldoketoreductase 1C2, (AKR1C1, encoding 20α-HSD), and *CYP11A1* [20]. This increase in steroidogenic enzyme expression in PCOS theca cells is selective, and does not include all of the enzymes involved in androgen and progestin biosynthesis, such as StAR or 17ß-hydroxysteroid dehydrogenase type V (17ß-HSDV) [20]. Both CYP17A1 [23] and CYP11A1 (Figs. 4–5) mRNA transcription and stability are augmented under basal conditions in PCOS theca cells. Also, the promoter elements required for increased transcriptional activity of the two promoters in PCOS theca cells map to sequences within −180 bp of the start site of transcription and contribute to significant basal regulation. As for mRNA stability, both *CYP11A1* and *CYP17A1* transcripts have greater that 2-fold longer half-life in PCOS theca cells. Moreover, *in vitro* studies suggest that sequences within the 5′-UTRs of both mRNAs are mandatory for differential transcript stability. Therefore, it seems likely that common regulatory mechanism(s) and/or signaling pathways control both transcription and mRNA stability of these transcripts, offering novel targets for treatment of excess androgens in women with PCOS.
